# Improving the functional expression of a *Bacillus licheniformis *laccase by random and site-directed mutagenesis

**DOI:** 10.1186/1472-6750-9-12

**Published:** 2009-02-23

**Authors:** Katja Koschorreck, Rolf D Schmid, Vlada B Urlacher

**Affiliations:** 1 Institute of Technical Biochemistry, Universitaet Stuttgart, Allmandring 31, 70569 Stuttgart, Germany

## Abstract

**Background:**

Laccases have huge potential for biotechnological applications due to their broad substrate spectrum and wide range of reactions they are able to catalyze. These include, for example, the formation and degradation of dimers, oligomers, polymers, and ring cleavage as well as oxidation of aromatic compounds. Potential applications of laccases include detoxification of industrial effluents, decolorization of textile dyes and the synthesis of natural products by, for instance, dimerization of phenolic acids. We have recently published a report on the cloning and characterization of a CotA *Bacillus licheniformis *laccase, an enzyme that catalyzes dimerization of phenolic acids. However, the broad application of this laccase is limited by its low expression level of 26 mg l^-1 ^that was achieved in *Escherichia coli*. To counteract this shortcoming, random and site-directed mutagenesis have been combined in order to improve functional expression and activity of CotA.

**Results:**

A CotA double mutant, K316N/D500G, was constructed by combining random and site-directed mutagenesis. It can be functionally expressed at an 11.4-fold higher level than the wild-type enzyme. In addition, it is able to convert ferulic acid much faster than the wild-type enzyme (21% vs. 14%) and is far more efficient in decolorizing a range of industrial dyes. The investigation of the effects of the mutations K316N and D500G showed that amino acid at position 316 had a major influence on enzyme activity and position 500 had a major influence on the expression of the laccase.

**Conclusion:**

The constructed double mutant K316N/D500G of the *Bacillus licheniformis *CotA laccase is an appropriate candidate for biotechnological applications due to its high expression level and high activity in dimerization of phenolic acids and decolorization of industrial dyes.

## Background

Laccases (benzenediol:oxygen oxidoreductases; EC 1.10.3.2.) belong to the family of multicopper oxidases, along with ascorbate oxidases and various ferroxidase enzymes. Laccases catalyze the four-electron reduction of molecular oxygen to water by the one-electron oxidation of four substrate molecules. These enzymes are able to utilize a broad range of substrates, including phenols, arylamines, anilines, thiols, and even some inorganic compounds [1-3]. The active center of laccases contains four copper ions that are conventionally classified into three types based on their coordination and spectroscopic properties [4]. The T1 site contains a single type 1 copper ion, which is tightly coordinated to a cysteine. The bond between the sulfur cysteine to copper is responsible for the characteristic blue color of laccase enzymes. The T2 site is a mononuclear center formed by type 2 copper, while two strongly coupled type 3 copper ions are located in the T3 site. Type 2 and type 3 copper ions form the trinuclear T2/T3 cluster, which binds and reduces molecular oxygen to water. Substrates are oxidized at the T1 site mediating the electron transfer to the T2/T3 cluster.

Laccases are widely distributed among fungi, higher plants [5], insects [6,7], and bacteria [8]. Fungal laccases are involved in lignin degradation [9], pigment production [10], and plant pathogenesis [11]. Plant laccases have a major role in the lignification process [12]. The physiological function of bacterial laccases is still unclear, but it is believed that they play a role in melanin production, spore coat resistance, morphogenesis and detoxification of copper [13].

Laccases are regarded as industrially relevant enzymes due to their broad substrate spectrum and wide range of reactions that they catalyze, including cross-linking of phenolic compounds, degradation of polymers, ring cleavage and oxyfunctionalization of aromatic compounds. These enzymes are particularly interesting as biocatalysts because they do not require costly cofactors like NADH or NADPH as many other oxidoreductases. Biotechnological applications of laccases include pulp-pretreatment in the paper production process, dye bleaching in the textile industry, detoxification of xenobiotics, organic synthesis, and bioremediation [14]. Potential fields of application lie in the synthesis of natural products like pigments and antioxidants through dimerization of phenolic and non-phenolic acids [15].

At present, fungal laccases are the only laccases used in industrial processes [16]. The use of bacterial laccases would open up new perspectives for biotechnological applications, because their expression level, activity and selectivity are far easier to improve by means of directed evolution compared to fungal laccases. Only a few bacterial laccases have so far been studied, although rapid progress in genome analysis suggests that these enzymes are widespread in bacteria [8]. The best-known bacterial laccase is CotA from *Bacillus subtilis*, an endospore coat protein exhibiting high thermostability [17]. Other bacterial laccases have been isolated from *Escherichia coli *(CueO) [18], *Bacillus halodurans *(Lbh-1) [19], *Thermus thermophilus *(TTC1370) [20], and several streptomycetes [21-24]. The crystal structures of two bacterial laccases, CotA and CueO, are well-studied [18,25] and site-directed mutagenesis has been used to examine the effect of several conserved amino acid residues on the structure, redox potential and activity of these enzymes [26-28]. While several reports describe the successful application of directed evolution methods for fungal laccases [29-34], random mutagenesis has so far not been used to improve the catalytic efficiency of bacterial laccases.

We have recently published a report on the cloning, expression and characterization of CotA (accession no. YP_077905), a new *Bacillus licheniformis *laccase, in *E. coli *[35]. CotA catalyzes the dimerization of mono- and bisubstituted 3-(4-hydroxyphenyl)-2-propenoic acids like sinapic acid or ferulic acid, and seems to be a promising candidate for the synthesis of natural products. However, the expression yield of active CotA in *E. coli *only amounts to 26 mg l^-1^.

Herein, random and site-directed mutagenesis methods were combined in order to improve the functional expression of CotA in *E. coli*. This led to three CotA mutants with a much higher expression level compared to the wild-type. The combination of the best mutations led to a double mutant with an even further improved functional expression, a higher activity in dimerization of ferulic acid, and a much better decolorization efficiency than the single mutants and wild-type.

## Results

### Construction and characterization of mutants

**Figure 1:**
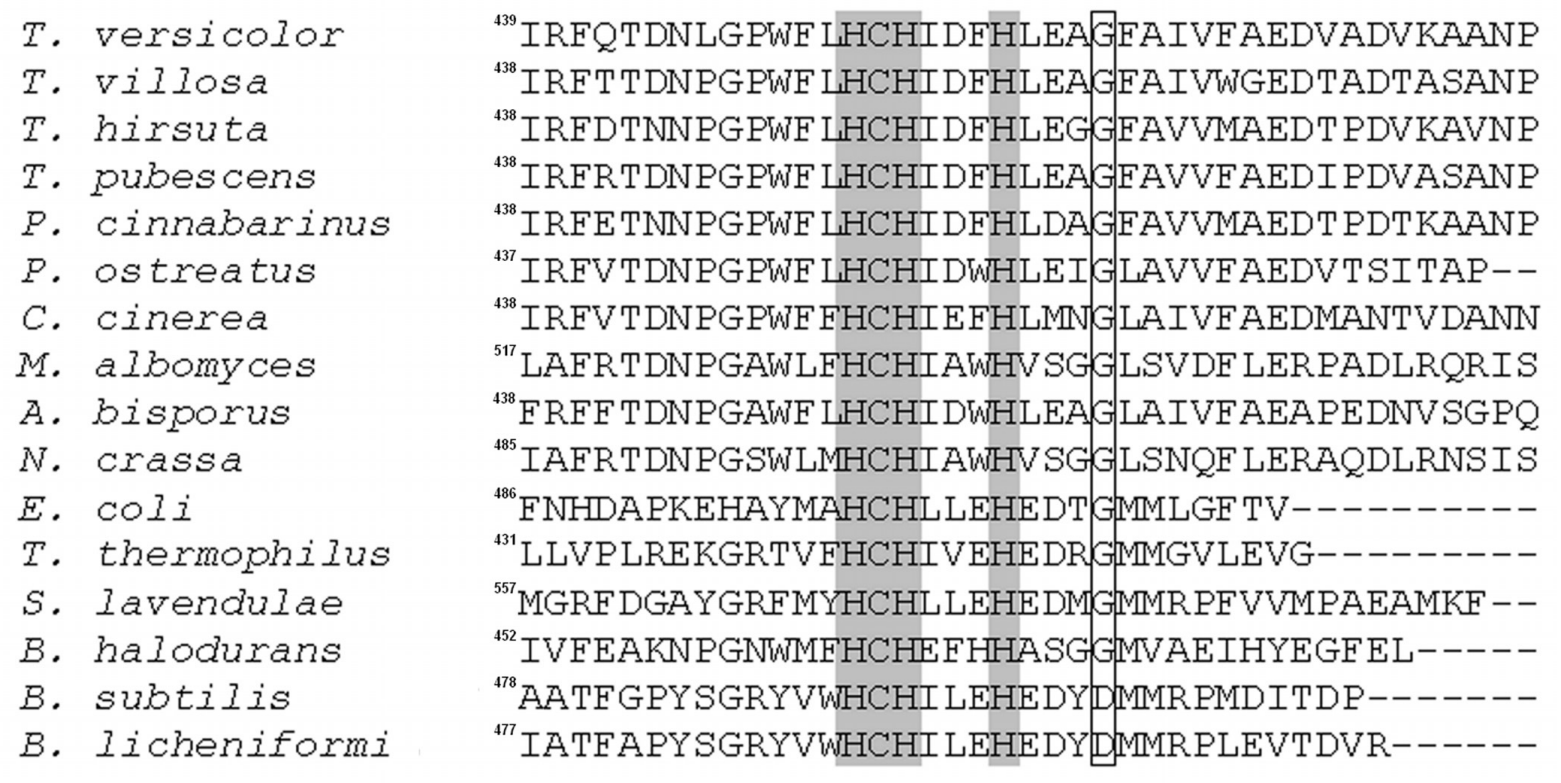
**C-terminal sequence alignment of fungal and bacterial laccase genes**. Highly conserved regions are represented against a gray background. The conserved glycine residue, corresponding to position 500 in the *B. licheniformis *CotA laccase, is framed.

Random mutations were introduced into the *B. licheniformis cotA *gene using error-prone PCR. 10,000 recombinant *E. coli *NovaBlue(DE3) clones were obtained. A mutation frequency of one to five mutations per laccase gene was determined by sequencing ten randomly selected individual CotA clones. From 6,000 clones analyzed, 38% had no activity towards 2,2'-azino-bis(3-ethylbenzothiazoline-6-sulfonic acid) (ABTS) at all despite a low mutation frequency. Twenty clones, which had an up to two-fold higher ABTS activity than wild-type, were selected and cultured in shaking flasks. The eight best clones, which had an activity increase of at least 90%, were sequenced. Each of these mutants possessed one to three amino acid substitutions. The mutations K316N and D500N were found in several clones. Judging from multiple sequence alignments aspartic acid is found only in the *B. licheniformis *CotA laccase (position 500) and in the *B. subtilis *CotA laccase (position 501). All other fungal and bacterial laccases examined had a glycine residue at the corresponding position (Figure 1). Position 316 in CotA is not very conserved among laccases, but several fungal and bacterial laccases had an asparagine residue at the corresponding position. In order to investigate the effect of each of these mutations on the expression level and the activity of *B. licheniformis *CotA, three single mutants, K316N, D500N, and D500G, were constructed. The volumetric activity, which was determined with ABTS, reached 1100 U l^-1 ^for K316N and 870 U l^-1 ^for D500N. This is considerably higher than that of wild-type CotA (410 U l^-1^). The replacement of Asp500 by glycine even resulted in a 5.4-fold higher volumetric activity (2200 U l^-1 ^for D500G). In addition, the double mutant K316N/D500G was constructed in order to study the combinatorial effect of both mutations on the laccase expression and activity. The volumetric activity of the double mutant was 8.3-fold higher (3400 U l^-1^) than that of wild-type CotA.

The investigation of kinetic properties of the purified CotA mutants revealed that the *K*_M _values of the mutants were only slightly higher than that of wild-type CotA and that the specific activities and catalytic efficiencies were lower (Table 1). It can therefore be assumed that the increase in volumetric activity was related to the increased functional expression of the mutant enzymes. The protein yield was calculated on the basis of volumetric and specific activity and led to an 11.4-fold increase in expression of the K316N/D500G double mutant in soluble active form compared to wild-type CotA. Approximately 300 mg of K316N/D500G can be obtained from 1 liter of culture.

**Table 1:** Catalytic parameters and expression levels of wild-type and CotA mutants.

Enzyme variant	*K*_M _(μM)^a^	Specific activity (μmol min^-1 ^mg^-1^)^a^	Catalytic efficiency (μmol min^-1 ^mg^-1 ^μM^-1^)	Volumetric activity (U l^-1^)^a^	Protein yield (mg l^-1^)^b^	Improvement factor
CotA	6.5 ± 0.2	16.0 ± 0.2	2.46	410 ± 10	26	1
D500G	9.5 ± 0.2	11.1 ± 0.1	1.17	2200 ± 200	199	7.8
K316N	10.0 ± 0.3	14.0 ± 0.2	1.40	1100 ± 100	79	3.1
K316N/D500G	8.4 ± 0.2	11.7 ± 0.2	1.39	3400 ± 100	291	11.4

^a ^Determined using ABTS as substrate in 0.1 M citrate/phosphate buffer, pH 4.0. Each value was calculated from three independent experiments.

^b ^Calculated on the basis of volumetric activity and specific activity of each laccase variant.

**Figure 2:**
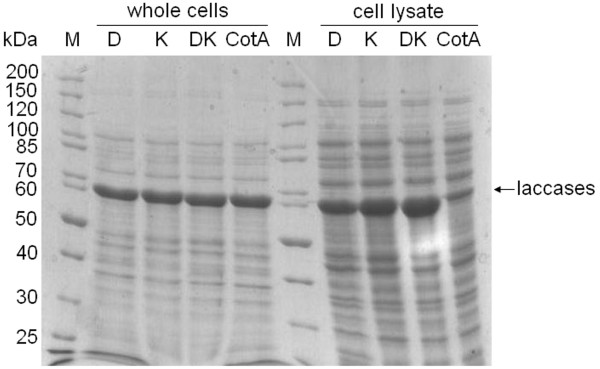
**SDS-PAGE analysis of constructed mutants and wild-type CotA**. Whole cells and cell lysates of the CotA mutants D500G (D), K316N (K), and K316N/D500G (DK) as well as of wild-type CotA (CotA) expressed overnight in *E. coli *BL21(DE3) cells; molecular size marker from Fermentas (M).

SDS-gel analysis showed that the overall expression of CotA mutants was similar to that of wild-type as equal quantities of laccases at ~65 kDa could be detected in whole cell extracts after overnight expression (Figure 2). However, the introduction of single or double K316N and D500G mutations in CotA led to a strong increase in the quantity of soluble CotA (see Figure 2 and Additional file 1). The UV-visible absorption spectrum of laccases has a broad band at 575–600 nm with a peak at 590 nm (corresponding to the T1 blue copper center). The CotA mutants and wild-type showed similar Abs_590 nm_/Abs_280 nm _ratios (~0.61), which is a characteristic for copper incorporation in the T1 site.

Since CotA is stable at elevated temperatures, the thermal stability of the mutants was studied at both 70°C and at room temperature. Between pH 5.0 and pH 7.0, wild-type and CotA mutants were of similar stability at both 70°C and room temperature. Independently of the temperature tested, the stability of all laccases decreased at pH 4.0, however residual activities of the mutants were significantly lower than the residual activity of wild-type CotA (data not shown).

The calculated pI value of the double mutant K316N/D500G and wild-type CotA was 6.81. The pI of K316N was slightly lower (6.74), and the pI of D500G was slightly higher (6.89) than that of wild-type.

### Oxidation of phenolic and non-phenolic acids

CotA catalyzes the dimerization of sinapic acid, ferulic acid, and caffeic acid as well as the oxidation of syringic acid to 2,6-dimethoxy-1,4-benzoquinone [35]. Several phenolic acids (sinapic acid, ferulic acid, caffeic acid, coumaric acid, syringic acid, and vanillic acid) and one non-phenolic acid (cinnamic acid) were tested in order to investigate the changes in the mutants' substrate specificity and activity. Neither wild-type nor the CotA mutants were able to oxidize coumaric acid, vanillic acid, and cinnamic acid (Table 2). Wild-type CotA had a higher activity for sinapic acid than the mutants, while K316N/D500G was more active in converting ferulic acid than wild-type (21% conversion vs. 14%). The mutations had nearly no effect on the activity of K316N/D500G in oxidation of caffeic and syringic acid. However, the activity of the single mutant K316N towards sinapic acid, ferulic acid, caffeic acid, and syringic acid was lower or similar to that of wild-type CotA, but always higher than that of D500G.

**Table 2:** Conversion of phenolic and non-phenolic acids by laccases after 90 min of reaction.

	Conversion after 90 min (%)						
Enzyme variant	Sinapic acid	Ferulic acid	Caffeic acid	Coumaric acid	Cinnamic acid	Syringic acid	Vanillic acid
CotA	49 ± 1.1	14 ± 0.6	18 ± 0.5	0	0	18 ± 0.6	0
D500G	31 ± 1.1	13 ± 0.6	10 ± 0.7	0	0	7 ± 0.9	0
K316N	39 ± 1.0	15 ± 0.6	14 ± 0.7	0	0	12 ± 0.7	0
K316N/D500G	45 ± 1.2	21 ± 0.6	20 ± 0.6	0	0	18 ± 0.6	0

Analysis of the reaction mixtures showed that the mutants produced the same products in the same ratios as wild-type CotA [35]. Syringic acid was oxidized to 2,6-dimethoxy-1,4-benzoquinone (*m/z *168). The main products of the laccases' reactions with sinapic acid, ferulic acid, and caffeic acid were dimers. The highest level of regioselectivity was obtained with sinapic acid, which was dimerized to dehydrodisinapic acid dilactone [36] (79%, *m/z *of 446) and two unknown products in a smaller quantity. Control reactions carried out in the absence of laccases did not lead to the oxidation of the compounds investigated.

### Decolorization of dyes

**Figure 3:**
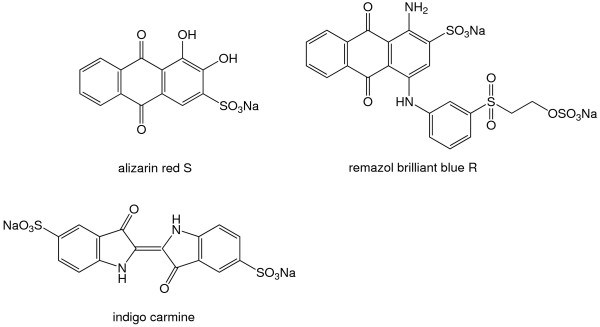
**Structures of the investigated dyes**.

**Figure 4:**
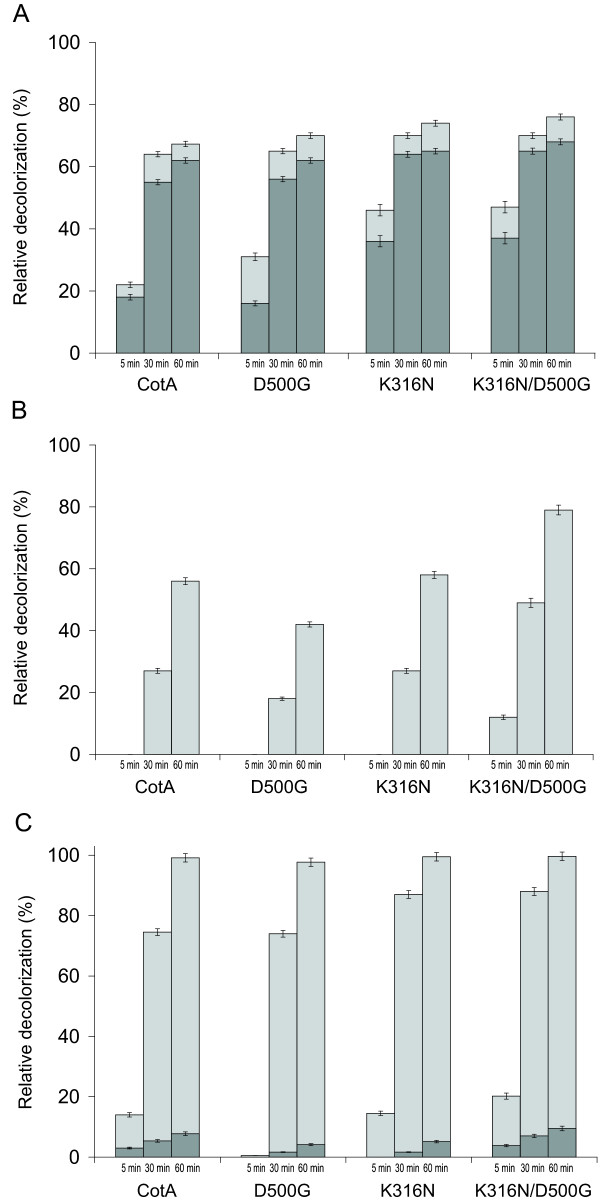
**Decolorization of different industrial dyes**. Decolorization catalyzed by wild-type CotA and CotA mutants was investigated in the absence (dark gray bars) and presence (light gray bars) of the redox mediator violuric acid after 5 min, 30 min, and 60 min of reaction. A) Decolorization of alizarin red S; B) decolorization of remazol brilliant blue R; C) decolorization of indigo carmine.

Wild-type CotA along with the K316N, D500G and K316N/D500G mutants were tested and compared for their ability to decolorize different industrial dyes in the presence and absence of the redox mediator violuric acid (Figure 3). Alizarin red S and remazol brilliant blue R (RBBR) (anthraquinone dyes) and indigo carmine (indigo dye) were decolorized by the laccases to a different extent (Figure 4). In general, the decolorization process was more efficient when violuric acid was present. The double mutant was more active than wild-type CotA and both single-mutants in bleaching of the three dyes. The activity of K316N and K316N/D500G towards alizarin red S was significantly higher (36% and 37%, respectively) than that of D500G and wild-type CotA (16% and 18% respectively) within the first 5 min of the decolorization reaction. The activities of the four laccases were quite similar after 30 and 60 minutes of reaction. Only a slight improvement in the reaction was achieved after adding the redox mediator violuric acid.

In contrast, RBBR was not decolorized at all when violuric acid was not added. In the presence of violuric acid, K316N/D500G displayed the highest decolorization activity (79% decolorization after 60 min). The activity of K316N was similar to that of wild-type CotA (58% and 56% decolorization after 60 min), whilst the activity of D500G was significantly lower (42% decolorization after 60 min).

It was possible to considerably improve the decolorization of indigo carmine by laccases upon addition of violuric acid and it reached almost 100% after 1 h of laccase treatment. After 30 min, K316N and K316N/D500G led to a significantly higher decolorization of indigo carmine in the presence of violuric acid (87% and 88%, respectively) than wild-type CotA or D500G (75% and 74%). The investigated dyes were not oxidized in the absence of laccase.

## Discussion

**Figure 5:**
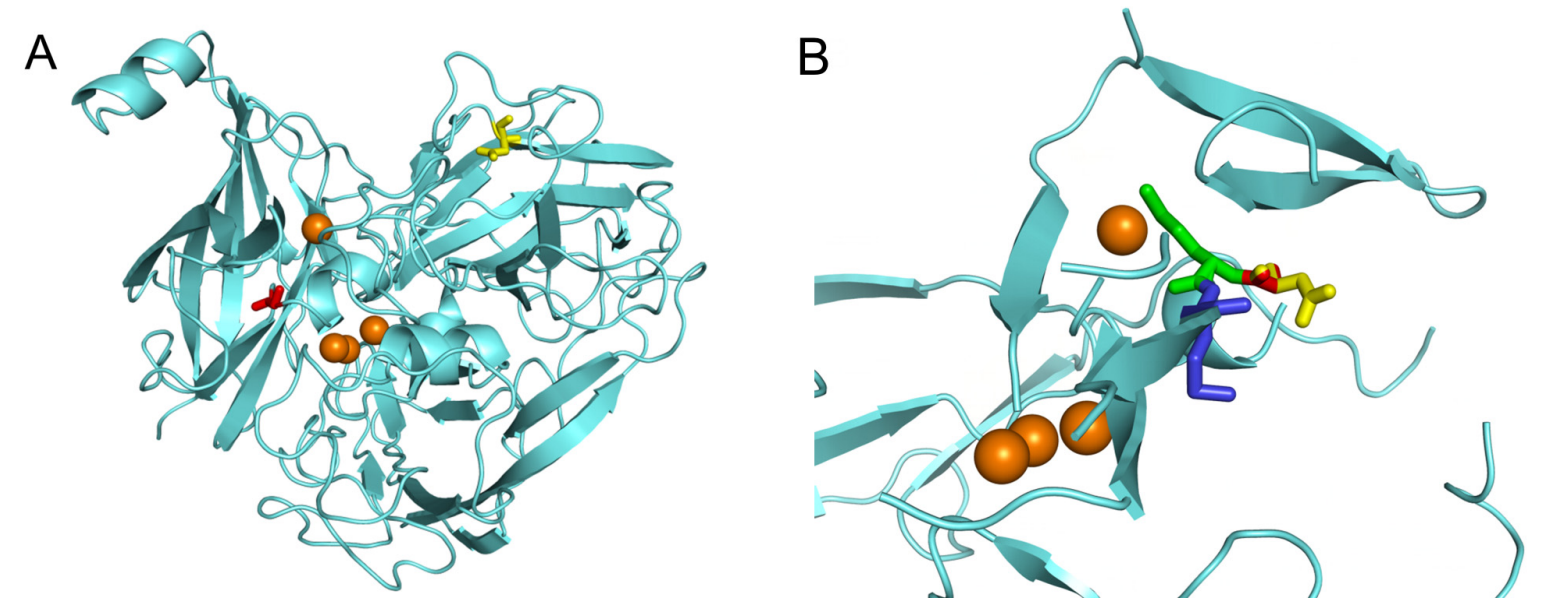
**Homology model of K316N/D500G (based on 1GSK **[25]). A) Amino acid residue asparagine 316 is shown in yellow; glycine 500 is shown in red. The T1 copper and the trinuclear copper cluster are highlighted in orange. B) Superposition of the catalytic center of wild-type CotA and K316N/D500G with Asp500 shown in yellow, Gly500 shown in red, the axial T1 ligand Met501 shown in green, and Met502 shown in blue. The T1 copper and the trinuclear copper cluster are highlighted in orange.

The random mutagenesis of *B. licheniformis *CotA resulted in 38% of completely inactive clones although only one to five mutations per gene were introduced. This observation is in line with other publications that reported that very few laccase positions can be mutated without a resultant loss of activity. This is due to highly conserved functionally essential regions of these enzymes [29]. Two mutations, K316N and D500N, were found in several clones with improved volumetric activity towards ABTS. Homology modeling was performed in order to rationalize the effect of these two positions. The homology models of wild-type CotA and K316N/D500G were based on the crystal structure of *B. subtilis *CotA laccase (PDB-ID 1GSK) [25], which possesses 63% sequence identity with wild-type *B. licheniformis *CotA. The amino acid residue at position 500 in *B. licheniformis *CotA is located in a loop region close to the T1 site (Figure 5A) and adjacent to methionine 501, which is the axial ligand of the T1 copper ion (Figure 5B). As has been reported previously, the replacement of the axial ligand by leucine or phenylalanine in *B. subtilis *CotA led to an increase in the redox potential of the corresponding mutants and had a negative effect on the catalytic constants [37]. As mentioned above, the amino acid at position 500 in *B. licheniformis *CotA is highly conserved in fungal and bacterial laccases and is usually a glycine. In our study, the substitution of aspartic acid at position 500 by glycine resulted in a larger space between Gly500 and Met502 (Figure 5B). This could entail structural changes at the T1 site as well as lead to more efficient folding of the enzyme and thus to a higher quantity of soluble protein. The latter has been observed for the D500G and K316N/D500G mutants. A structural change at the T1 site might also explain the reduced catalytic activity of the D500G mutant towards all substrates tested.

Position 316 is located at the end of a β-strand on the surface of the protein, far from position 500 and the copper centers. Nevertheless, it points into the interior (Figure 5A). In some bacterial and fungal laccases, including the investigated laccase, this position is occupied by asparagine. However, proline, aspartic acid, glutamine, leucine, alanine, and threonine residues have also been found at the corresponding positions in other laccases. Although this position mainly seems to have an effect on the catalytic activity of the enzyme, in combination with D500G the mutation K316N resulted in a significant increase in the quantity of soluble active CotA laccase. Up to 300 mg of K316N/D500G can be obtained from 1 liter culture in shaking flasks. This is much higher than the yields achieved for fungal laccases in *Pichia *[38-40] and similar to the strongly expressed *Melanocarpus albomyces *laccase in *Trichoderma reesei *[41].

It is interesting to note that the combination of the mutations K316N and D500G resulted in a more active enzyme relative to single mutants. The specific activity of K316N/D500G towards ferulic and caffeic acid was higher than that of the wild-type enzyme as well. Furthermore, the double mutant K316N/D500G had a significantly higher specific activity towards alizarin red S, RBBR, and indigo carmine than the single mutants or wild-type CotA. Since the decolorization activity of K316N was higher than that of D500G and wild-type it can be safely assumed that it is mutation K316N that contributes to an increase in the double mutant's catalytic activity for these dyes. The addition of a redox mediator significantly improved the decolorization of the textile dyes, as has also been shown by other authors [42,43]. The highest decolorizing activity in the presence of the redox mediator violuric acid was once again achieved with the double mutant K316N/D500G, which makes it an appropriate candidate for biotechnological applications. The synergistic effect of the two mutations, which resulted in a laccase enzyme with higher activity and strongly improved functional expression was not foreseen by the homology model. This clearly demonstrates the importance of random mutagenesis for the development of more efficient biocatalysts.

## Conclusion

In summary, the combination of random and site-directed mutagenesis resulted in the *B. licheniformis *CotA double mutant K316N/D500G. This mutant had an 11.4-fold higher expression level in functional soluble form, a higher ferulic acid dimerization activity and a greater efficiency in the decolorization of industrial dyes compared to wild-type CotA. The analyses showed that glycine at position 500 had a major impact on the increase of the enzyme's functional expression in *E. coli*. However, glycine also reduces the specific activity of the enzyme. Asparagine at position 316 resulted in both, higher functional expression and improved specific activity for different dyes. It is interesting to note that the synergistic effect of both mutations on the enzyme's properties could not be predicted from their individual effects.

Laccases are very attractive enzymes for application in green chemistry, because they use molecular oxygen and produce only water as a by-product. In addition, they do not require NADH or NADPH, costly cofactors that are required by many other oxidoreductases. The high expression level and enhanced activity of K316N/D500G offers for the first time the opportunity to use a bacterial laccase for biotechnological applications.

## Methods

### Materials, *E. coli *strains, and plasmids

All chemical reagents were of analytical grade or higher and were purchased from Sigma-Aldrich (Deisenhofen, Germany). Enzymes were obtained from Fermentas (St. Leon-Rot, Germany) or New England Biolabs (Massachusetts, USA). *Escherichia coli *strain DH5α (F^- ^φ*80lacZΔM15 *Δ*(lacZYA-argF)U169 deoR recA1 endA1 hsdR17*(r_k_^- ^m_k_^+^) *supE44 thi-1 gyrA96 relA1*) was purchased from Clontech (Heidelberg, Germany). *E. coli *strains BL21(DE3) (F^- ^*dcm ompT hsdS*(r_B_^- ^m_B_^-^) *gal *(DE3)) and NovaBlue(DE3) (*endA1 hsdR17*(r_K12_^- ^m_K12_^+^) *supE44 thi-1 recA1 gyrA96 relA1 lac *(DE3) F' [*proA*^+ ^*B*^+ ^*lacI*^*q*^*ZΔM15*::Tn*10*] (Tet^r^)) as well as the pET22b+ vector were obtained from Novagen (Madison, USA). The GeneMorph II random mutagenesis kit was purchased from Stratagene (La Jolla, CA, USA).

### Library construction

Random mutations were introduced into the CotA encoding gene of *Bacillus licheniformis *at low frequency [35] using the GeneMorph II random mutagenesis kit. Error-prone PCR was performed with the following primers: CotA_forward (5'-CTAGTGA*CATATG*AAACTTGAAAAATTCGTTGACCGG-3') and CotA_reverse (5'-CGTC*GAATTC*TTATTGATGACGAACATCTGTCACTTC-3'). The recognition sites for *Nde*I and *Eco*RI endonucleases are italicized. The following PCR conditions were used: 95°C (2 min, once), 95°C (1 min), 53°C (1 min), 72°C (2 min), 30 cycles and 72°C (10 min, once). The resulting PCR fragment was digested with *Nde*I and *Eco*RI and ligated in the corresponding sites of the linearized and dephosphorylated pET22b+ vector. *E. coli *NovaBlue(DE3) cells were transformed with the resulting plasmids.

### Library screening

Single clones were picked into 96-well plates with the help of a picking robot (Biopick automated colony picking system, Biorobotics, Woburn, USA). The plates contained 150 μl Terrific-Broth (TB) medium with 5% dimethyl sulfoxide (DMSO) and 100 μg ml^-1 ^ampicillin. The plates were incubated at 37°C and shaken at 600 rpm (MS1 minishaker, IKA, Staufen, Germany) for 24 h and stored at -80°C (master plates). New 96-well plates were inoculated from the master plates and incubated at 37°C and 600 rpm for 24 h. 96-Well deep-well plates containing 600 μl TB medium supplemented with ampicillin were inoculated with the new cultures and incubated for 8 h at 37°C and 600 rpm. Expression was induced by adding 100 μl induction solution (TB medium, containing 1.75 mM isopropyl β-D-thiogalactopyranoside (IPTG), 14 mM CuSO_4 _and 100 μg ml^-1 ^ampicillin). Cells were incubated at 25°C and 400 rpm for an additional 24 h. The plates were then centrifuged for 20 min at 3,220 *g*, 4°C and the pellets were subsequently stored at -80°C. For cell lysis, the pellets were resuspended in 600 μl resuspension buffer (50 mM potassium phosphate buffer, pH 7.5 containing 1 mg ml^-1 ^lysozyme, 0.05 U ml^-1 ^DNase and 10 mM MgSO_4_) and incubated for 45 min at 37°C. The cell debris was removed by centrifugation for 30 min at 3,220 *g*, 4°C. A laccase activity assay was performed by transferring 150 μl of lysate to a new 96-well plate with the help of a pipetting robot (Janus Automated Workstation (PerkinElmer)). 50 μl assay solution (0.1 M citrate/phosphate buffer, pH 5.0 containing 5 mM 2,2'-azino-bis(3-ethylbenzothiazoline-6-sulfonic acid) (ABTS) was added to each well. Plates were incubated for 2 min at room temperature and absorption at 420 nm (ε_420 _= 36,000 M^-1 ^cm^-1^) was recorded on a microtiter plate spectrophotometer (Spectramax 340 PC, Molecular Devices, Sunnyvale, USA).

### Site-directed mutagenesis

The mutants K316N, D500N, D500G and K316N/D500G were constructed by PCR using the QuikChange™ site-directed mutagenesis kit from Stratagene according to the manufacturer's protocol. Mutations were introduced using the following primers: K316N_forward 5'-GTGACACTGAAAAACCGGATCGGCTGC-3'; K316N_reverse 5'- GCAGCCGATCCGGTTTTTCAGTGTCAC-3'; D500N_forward 5'- CACGAAGATTACAATATGATGCGCCC-3'; D500N_reverse 5'- GGGCGCATCATATTGTAATCTTCGTG-3'; D500G_forward 5'- CACGAAGATTACGGTATGATGCGCCC-3', and D500G_reverse 5'- GGGCGCATCATACCGTAATCTTCGTG-3'.

### Expression and purification of mutants

*E. coli *BL21(DE3) cells carrying pET22cotA, pET22K316N, pET22D500N, pET22D500G or pET22K316N/D500G were grown in 5 ml Luria-Bertani (LB) medium supplemented with 100 μg ml^-1 ^ampicillin at 37°C and 180 rpm for 5 h. 500-ml shaking flasks containing 50 ml Terrific Broth (TB) medium supplemented with ampicillin were then inoculated with 0.5 ml of the prepared culture and incubated at 37°C and 180 rpm. Expression was induced at an optical density (OD_578_) of 1.8 by adding 0.25 mM IPTG and 2.0 mM CuSO_4_. The cells were incubated overnight at 18°C in a shaking incubator (140 rpm). The culture was harvested by centrifugation (20 min, 8,000 *g*, 4°C) and the pellets were resuspended in 50 mM potassium phosphate buffer, pH 7.5 containing 0.1 mM phenylmethylsulfonyl fluoride (PMSF) and 0.3 mM CuSO_4_. The cells were disrupted by sonification on ice and the cell debris was removed by centrifugation (30 min, 8,000 *g*, 4°C). Subsequently, the supernatant was incubated for 15 min at 70°C and the denatured proteins were removed by centrifugation (10 min, 10,000 *g*, 4°C). Purification was performed on an Äkta explorer FPLC system (Amersham Biosciences, UK). 10 ml laccase solution was loaded on a 22 ml Q-Sepharose FF (Amersham Biosciences) column. The column was washed with three column volumes of 50 mM potassium phosphate buffer, pH 7.5. The laccase enzyme was eluted from the column with 250 mM sodium chloride in 50 mM potassium phosphate buffer, pH 7.5. Fractions containing laccase activity (as measured using the ABTS assay) were pooled, concentrated, and desalted by Amicon ultrafiltration (membrane cut-off 10 kDa, Millipore, USA). Protein concentration was determined using a Bradford assay [44]. Bovine serum albumin was used as standard. SDS-polyacrylamide gel electrophoresis (SDS-PAGE) was performed in a Minigel-Twin (Biometra, Goetttingen, Germany) using a 10% polyacrylamide running gel [45].

### Laccase activity assay

Laccase activity was assayed at room temperature using ABTS. The assay mixture contained 0.5 mM ABTS and 0.1 M citrate/phosphate buffer, pH 4.0. The oxidation of ABTS led to an absorbance increase at 420 nm (ε_420 _= 36,000 M^-1 ^cm^-1^). One unit is defined as the amount of enzyme that oxidizes 1 μmol of substrate per min. The assay was performed in triplicate.

### Characterization of laccases

Kinetic parameters of purified enzymes were determined at room temperature using the ABTS assay with concentrations of 5–500 μM ABTS. The data were fitted to the Michaelis-Menten equation by linear regression. The thermal stability of the laccase enzymes was measured at 70°C by incubating the enzyme solutions (0.2 mg ml^-1^) in 0.1 M citrate/phosphate buffer, pH 4.0, 5.0 and 7.0, respectively. After 30 min, samples were withdrawn, cooled, and the residual activity was determined using the ABTS assay. Stability at room temperature was determined by incubating 0.8 mg ml^-1 ^laccase solution in 0.1 M citrate/phosphate buffer, pH 4.0, 5.0 and 7.0, respectively. Residual activity was measured after 24 h using the ABTS assay. All reactions were performed in triplicate. The standard deviations were less than 5%.

### Oxidation of phenolic and non-phenolic acids

All experiments were performed in 25-ml round flasks containing 50 μg purified laccase in 10 ml 0.1 M citrate/phosphate buffer, pH 5.0. Sinapic acid, ferulic acid, caffeic acid, coumaric acid, cinnamic acid, syringic acid, and vanillic acid, respectively, were dissolved in 100 μl DMSO and added to the reaction, producing a final concentration of 5 mM. Reactions were performed at room temperature for 90 min. Reaction progress was followed by taking samples during the course of the reactions. The samples were acidified to pH 2–3 with 6 M HCl, extracted with ethyl acetate, evaporated and redissolved in acetonitrile. They were analyzed using high-performance liquid chromatography (HPLC) and the educts and products of the reactions were identified by LC/MS analysis as described elsewhere [35]. All reactions were performed in triplicate. Substrate conversion was calculated based on the ratio of the detected peak areas of substrates and products in HPLC chromatograms after 90 min of reaction.

### Decolorization of dyes

Three dyes, alizarin red S, remazol brilliant blue R (RBBR), and indigo carmine were used to evaluate the ability of wild-type and CotA mutants to decolorize industrial dyes. The enzymatic treatment of dyes was performed in the presence or absence of 1 mM redox mediator (violuric acid) in 1 ml 0.1 M citrate/phosphate buffer, pH 5.0 containing 0.04 mg ml^-1 ^of dye and 10 μg of purified laccase. The reaction mixtures were incubated at room temperature and the decolorization activity of laccases was determined spectrophotometrically as the relative decrease of absorbance. The test wavelengths for alizarin red S, RBBR, and indigo carmine were 423 nm, 592 nm, and 595 nm, respectively, the absorbance maximum of the dyes. All reactions were performed in triplicate.

## Authors' contributions

KK participated in the design of the study, carried out the experiments and drafted the manuscript. RDS conceived the study. VBU participated in the design of the study and in writing the manuscript. All authors read and approved the final manuscript.

## Supplementary Material

Additional file 1 **SDS-PAGE analysis of pellet (insoluble) fraction of CotA mutants and wild-type.** Pellet fractions of the CotA mutants D500G (D), K316N (K), and K316N/D500G (DK) as well as of wild-type CotA (CotA) expressed overnight in *E. coli *BL21(DE3) cells; molecular size marker from Fermentas (M). Click here for file
