# Supplementary figures and images for: Improving the functional expression of a Bacillus licheniformis laccase by random and site-directed mutagenesis
Source: BMC Biotechnol. 2009 Feb 23;9:12. doi: 10.1186/1472-6750-9-12 (PMC2653023; doi:10.1186/1472-6750-9-12)

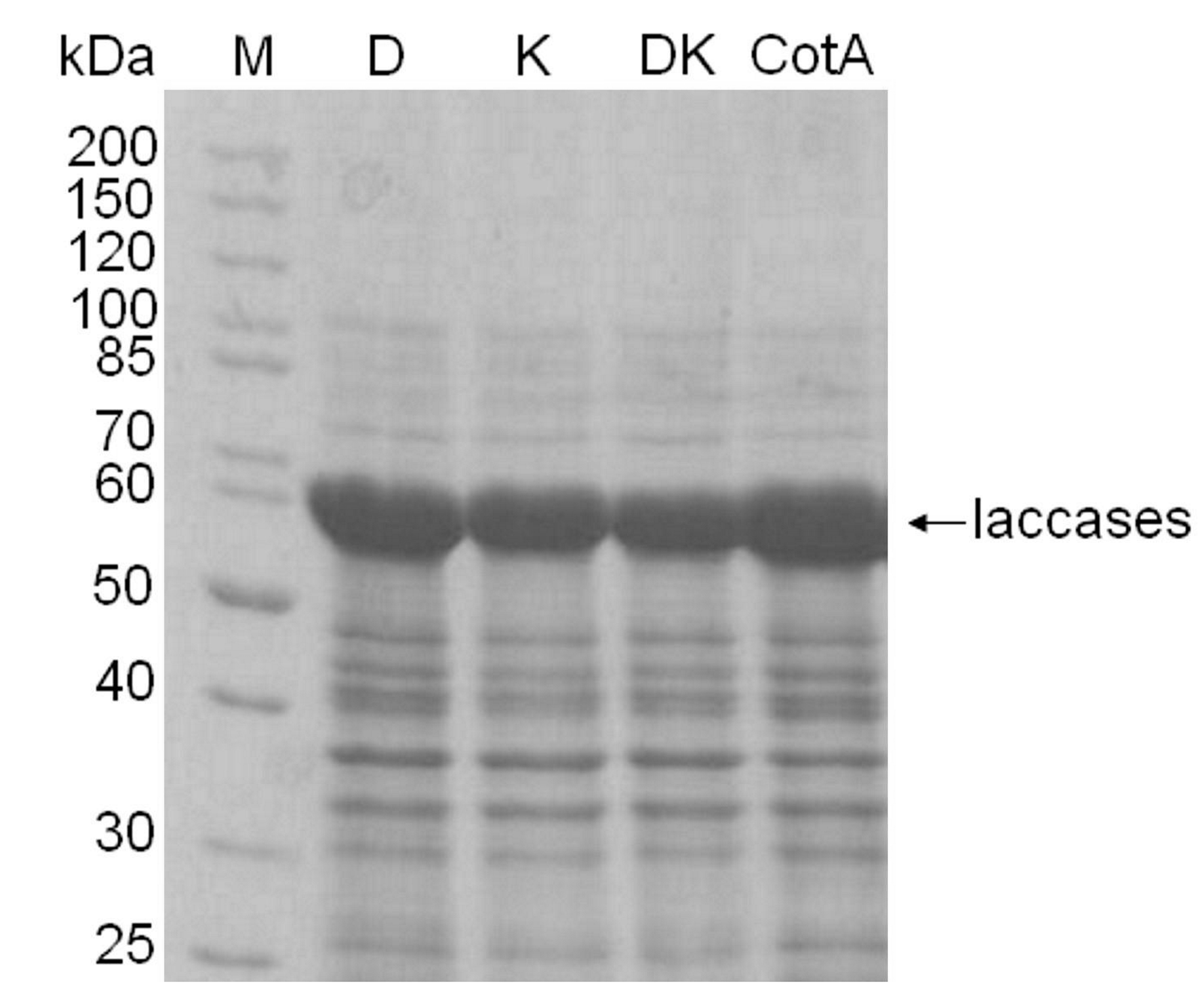

Supplement: Additional file 1 — SDS-PAGE analysis of pellet (insoluble) fraction of CotA mutants and wild-type. Pellet fractions of the CotA mutants D500G (D), K316N (K), and K316N/D500G (DK) as well as of wild-type CotA (CotA) expressed overnight in E. coli BL21(DE3) cells; molecular size marker from Fermentas (M). [file 1472-6750-9-12-S1.png]
